# Pectoral nerves (PECS) and intercostal nerve block for cardiac resynchronization therapy device implantation

**DOI:** 10.1186/2193-1801-3-409

**Published:** 2014-08-05

**Authors:** Atsushi Fujiwara, Nobuyasu Komasawa, Toshiaki Minami

**Affiliations:** Department of Anesthesiology, Osaka Medical College, Daigaku-machi 2-7, Takatsuki, Osaka, 569-8686 Japan

**Keywords:** Cardiac resynchronization therapy device implantation, Pectoral nerves (PECS) block, Intercostal nerve block

## Abstract

A 71-year-old man was scheduled to undergo cardiac resynchronization therapy device (CRTD) implantation. He was combined with severe chronic heart failure due to ischemic heart disease. NYHA class was 3 to 4 and electrocardiogram showed non-sustained ventricular. Ejection fraction was about 20% revealed by transthoracic echocardiogram. He was also on several anticoagulation medications. We planned to implant the device under the greater pectoral muscle. As general anesthesia was considered risky, monitored anesthesia care utilizing peripheral nerve block and slight sedation was scheduled. Pectoral nerves (PECS) block and intercostal block was performed under ultrasonography with ropivacaine. For sedation during the procedure, continuous infusion of dexmedetomidine without a loading dose was performed. The procedure lasted about 3 hours, but the patient showed no pain or restlessness. Combination of PECS block and intercostal block may provide effective analgesia for CRTD implantation.

## Background

Effective analgesia and sedation during cardiac resynchronization therapy device (CRTD) implantation is important from the perspectives of surgical procedure and circulation stability (Looi et al. 2013).

The pectoral nerves (PECS) block is relatively easy and reliable peripheral nerve block which was inspired by the transversus abdominis plane block and the brachial plexus block with infraclavicular approach (Blanco 2011). PECS block targets the lateral and median pectoral nerves at an interfascial plane between the pectoralis major and minor muscles (Porzionato et al. 2012). The main indications are breast surgery by tissue expanders or subpectoral prosthesis because the distension of these muscles is rather painful (Blanco 2011) (Blanco et al. 2012).

Here we report the successful application of pectoral nerves (PECS) and intercostal nerve block for CRTD implantation.

### Case presentation

A 71-year-old man (height, 154 cm; weight, 41 kg) was scheduled to undergo CRTD implantation. He suffered from chronic heart failure due to ischemic heart disease. NYHA class was 3 to 4 and nonsustained ventricular tachycardia was observed. Transthoracic echocardiography showed an ejection fraction of 20%. He was also on several anticoagulation medications. We planned to implant the device under the greater pectoral muscle. As general anesthesia was considered risky, monitored anesthesia care with peripheral nerve block and slight sedation was scheduled.

Intercostal nerve block at the level of the first and second intercostal space was performed under ultrasonography with 4 ml of 0.375% ropivacaine. With linear ultrasound probe positioning similar place when performing an infraclavicular brachial plexus block. After identifying the pectoralis major muscle, we checked the location of the pectoral branch of the thoracio-acromial artery between the pectoralis muscles with colour Doppler. Then, we placed 10 ml of 0.375% ropivacaine into the interfascial plane between pectoralis major and minor muscles (Blanco 2011). The puncture point of the PECS block needle was 2–3 cm from the surgical point.

For sedation during the procedure, continuous infusion of 0.4 μg・kg^-1^・h^-1^ dexmedetomidine without a loading dose was performed. The procedure lasted about 3 hours, but the patient showed no pain or restlessness. Also, no significant hemodynamic change such as heart rate and blood pressure was admitted (within 10 percent).

## Discussion and evaluation

Analgesia and sedation during CRTD implantation is often challenging, as systemic administration of sedatives and analgesics can disturb circulation (Looi et al. 2013). PECS block is a relatively new peripheral nerve block strategy for breast surgery pain management (Blanco 2011) (Blanco et al. 2012). With PECS and Intercostal nerve block, we can not only provide effective analgesia for relatively long procedures but also avoid the risk of local anesthetic intoxication. Too much local anesthetic administration may lead to the insufficient wound healing, local anesthetic intoxication leading to patient stress (Butterworth 2010). With PECS and intercostal nerve block, we could minimize the dose of local anesthetics under the guide of ultrasonography. However, as there is a risk of peripheral nerve injury by the block needle, we should pay thorough attention during the procedure.

We utilized dexmedetomidine as the sedatives during the procedure. Effect of dexmedetomidine to circulatory and respiratory suppression is relatively smaller compared to propofol (Curtis et al. 2013). Combination of peripheral nerve block and dexmedetomidine sedation may be beneficial for monitored anesthesia care in patients with severe cardiopulmonary problems.

## Conclusion

Our findings suggest that a combination of PECS block and intercostal nerve block can provide effective analgesia for CRTD implantation.

### Patient consent

Written consent was obtained from the patient for the publication of this report.
